# Tobacco Ointment in Rigid Cervix

**Published:** 1893-08

**Authors:** 


TOBACCO OINTMENT IN RIGID CERVIX.—Dr. T. F. Verbeck, in *Chicago Medical Times*, highly recommends the application of this remedy in cases of rigid os uteri. He has repeatedly used this ointment, thoroughly applying with the finger over the cervix as high as vaginal insertion, and has always noted an immediate change in the condition of the parts, and a most satisfactory and complete dilatation within half an hour No unpleasant symptoms followed its application, but it decidedly relieved the irritative "nagging" pains which gave way to normal expulsive contractions.

